# Novel *ABCG8* Mutation in Pediatric Sitosterolemia: A Case Report of Siblings with Hemolytic Anemia

**DOI:** 10.1016/j.htct.2026.106472

**Published:** 2026-05-23

**Authors:** Subhrakamal Saha, Tuphan Kanti Dolai, Kaustav Ghosh, Ekta Jajodia

**Affiliations:** a Department of Hematology, Nilratan sircar medical college and hospital, Kolkata, West Bengal, India; b Unipath speciality laboratory, Ahmedabad, Gujarat, India

## Introduction

Sitosterolemia, also referred to as phytosterolemia (MIM #210,250), is a rare autosomal recessive sterol storage disorder first identified in 1974 by Bhatacharyya & Connor [1]. It results from pathogenic mutations in the *ABCG5* and *ABCG8* genes, which encode sterolin-1 and sterolin-2, respectively. These proteins form a heterodimeric transporter complex responsible for the efflux of dietary plant sterols from enterocytes back into the intestinal lumen and from hepatocytes into the bile, therefore limiting systemic absorption of plant sterols [2]. The hematological phenotype of sitosterolemia was historically described as Mediterranean stomatocytosis with thrombocytopenia [3]. Due to underrecognition and limited access to diagnostic assays, the actual prevalence remains unknown, although the condition is believed to be extremely rare [4]. Sitosterolemia has marked clinical heterogeneity, which often results in delayed diagnosis. Clinical manifestations can range from cutaneous xanthomas, premature atherosclerotic cardiovascular disease, and arthritis to endocrine abnormalities such as thyroid dysfunction. Hematologic features are also common including unexplained hemolytic anemia, macrothrombocytopenia, splenomegaly, and bleeding tendencies [5]. As routine lipid profiles do not measure plant sterols, diagnosis often requires high clinical suspicion and genetic or sterol quantification testing. Here, we report two pediatric siblings with hemolytic anemia and macrothrombocytopenia, ultimately diagnosed with sitosterolemia through genetic analysis.

## Case presentations

### Case 1

**Fig. 1:**
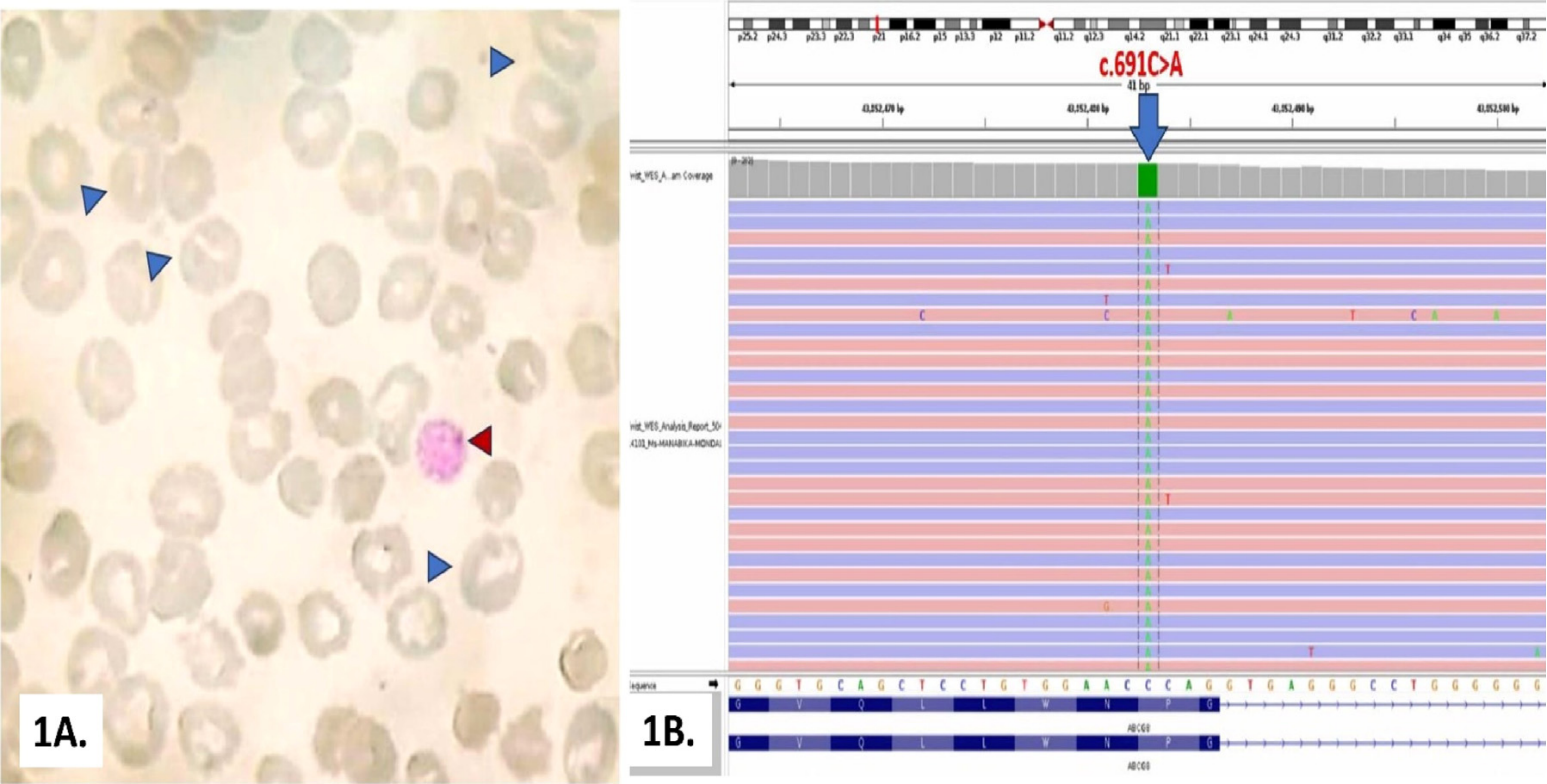
**A)** Peripheral blood smear (Leishman Giemsa stain, 100x) of a 12-year-old girl with hemolytic anemia showing stomatocytes (blue arrow head) and large platelet (red arrow head); **B)** Integrative genomics viewer visualization of alignments in chromosome 2 is showing C > A variation corresponding to *ABCG8*:c.691C>A;p.(Pro231Thr).

A 12‑year‑old female, the product of a non‑consanguineous union, had reported a three‑year history of intermittent jaundice and pallor. The patient has a transfusion history of four units of packed red blood cells (RBCs) over a two years span but no history of fever, rash, bleeding, joint pain or skin lesions. The physical examination demonstrated moderate pallor with mild splenomegaly (2 cm below the left costal margin) without any lymphadenopathy. A complete blood count showed anemia (hemoglobin [Hb] 7.6 *g*/dL) with corrected reticulocyte count of 9 %, indicating active hemolysis. Total leukocyte count measured 7600/µL with an absolute neutrophil count of 5320/µL and thrombocytopenia (80,000/µL). RBC indices showed increased Mean Corpuscular Volume (MCV) of 102.5 fl with normal Mean Corpuscular Hemoglobin (MCH), Mean Corpuscular Hemoglobin Concentration (MCHC) and Red Cell Distribution Width (RDW). A peripheral smear revealed stomatocytes and macrothrombocytopenia (Figure 1A), suggestive of an underlying red‑cell membrane defect. Iron profile, ferritin, vitamin B12 and folate were normal, ruling out iron overload or nutritional deficiency. Laboratory studies revealed an elevated LDH of 572 IU/L (range: 140–280 IU/L) and indirect hyperbilirubinemia (2 mg/dL). Serum urea, creatinine and the standard lipid profile were within reference limits. Hemoglobin High-Performance Liquid Chromatography was normal and Direct Coombs test (DCT), Paroxysmal Nocturnal Hemoglobinuria testing by Fluorescent Aerolysin, and an Eosin-5′-Maleimide Binding Test were negative, therefore ruling out other causes of hemolytic anemia. A bone‑marrow aspirate demonstrated normocellular marrow with erythroid hyperplasia, with concordant findings in a bone marrow biopsy. Given the constellation of stomatocytic hemolytic anemia and presumed autosomal‑recessive inheritance, targeted next‑generation sequencing was performed which identified a novel homozygous missense variant in *ABCG8*:c.691C>A; p.(Pro231Thr) in exon 5 (Figure 1B). The mutation was pathogenic and was concordant with sitosterolemia. Management comprised dietary restriction of plant sterols and initiation of ezetimibe (10 mg daily). The subsequent follow‑up documented normalization of Hb levels, resolution of hyperbilirubinemia, and obviated the need for transfusional support.

### Case 2

The 10-year-old younger sibling of Case 1 underwent evaluation as part of a familial screening protocol following the elder sister’s diagnosis of sitosterolemia. She was clinically asymptomatic, with no history of jaundice, bleeding tendencies, or pallor requiring blood transfusions. A physical examination showed mild pallor with no hepatosplenomegaly, lymphadenopathy or xanthomas. Hematological investigations revealed mild anemia (Hb: 9.9 g/dL) with a corrected reticulocyte count of 4%. The MCV was 95.3 fL, with normal MCH, MCHC and RDW. The total leukocyte count was 6100/µL with platelet count mildly reduced (105,000/µL). A peripheral blood smear showed normocytic normochromic red blood cells with occasional stomatocytes. Serum LDH was modestly elevated (480 IU/L), and bilirubin levels were mildly raised (total: 1.6 mg/dL; direct: 0.5 mg/dL). A DCT was negative and a nutritional workup (iron, vitamin B12, folate) was non-contributory. Molecular analysis detected the same homozygous missense mutation - *ABCG8*:c.691C>A; p.(Pro231Thr) - in exon 5 as was observed in the index case, confirming the diagnosis of sitosterolemia. Despite being asymptomatic, she was initiated on a plant sterol-restricted diet and enrolled in a regular follow-up program. Subsequent evaluations showed stable hematologic parameters and no development of clinical symptoms.

Hematological Parameters and six-month follow-up data of both siblings are summarized in Table 1.

**Table 1:** Hematological Parameters and six-month follow-up of two siblings with Sitosterolemia.

Parameter	Case 1 (12-year-old female)	Case 1 Follow-up	Case 2 (10-year-old female)	Case 2 Follow-up	Reference Range
**Hemoglobin (g/dL)**	7.6	11.5	9.9	11.2	11.5–15.5
**Mean corpuscular volume (fL)**	102.5	95	95.3	94	80–100
**Reticulocyte count (%)**	9	2	4	2	0.5–2
**Platelets (/µL)**	80,000	130,000	105,000	160,000	150,000–450,000
**Serum lactate dehydrogenase (IU/L)**	572	220	480	260	140–280
**Total Bilirubin (mg/dL)**	2.0	0.8	1.6	0.9	0.3–1.2
**Peripheral smear**	Stomatocytes, macrothrombocytopenia	Normalized morphology	Occasional stomatocytes	Normal morphology	–
**Therapy**	Plant sterol restriction +ezetimibe 10 mg/day	Continued	Plant sterol restriction	Continued	–

## Discussion

Sitosterolemia is a rare autosomal recessive disorder of sterol trafficking that remains underrecognized due to its heterogeneous clinical spectrum. Notably, its presentation as hemolytic anemia is even rarer, often mimicking other congenital or immune-mediated hematologic disorders. While roughly 200 cases of sitosterolemia have been documented to date, emerging genetic data suggest the condition is likely underdiagnosed. The estimated frequency of heterozygous loss-of-function variants of the *ABCG5* or *ABCG8* genes is approximately 1 in 220 individuals, suggesting that the prevalence of sitosterolemia caused by homozygous or compound heterozygous mutations could be as high as 1 in 200,000 [6].

### Demographic distribution

Both the siblings in the present report are females aged 12 and 10 years. The cohort by Wang et al. [7] comprised adults (aged 23–61 years; n = 13) with a near-equal male-to-female ratio (1:1.16). Desai et al. [8] reported younger patients (aged 7–29 years; n = 4), while Risinger et al. [9] and Zhao et al. [10] reported cases of a 19-year-old male and an eight-year-old male, respectively. The lack of sex predilection across all cases suggests autosomal inheritance, but pediatric-onset cases may display earlier and more severe hematologic manifestations due to prolonged exposure to sterol imbalance.

### Genotypic spectrum and phenotypic implications

Wang et al. [7] observed both nonsense and frameshift variants including C18802T, C20896T, and ISV713G>A. Desai et al. [8] identified predominantly truncating variants in *ABCG5* (e.g., p.R243X, p.R408X). The mutation (p.Arg446Ter in *ABCG5*) reported by Zhao et al. [10] reaffirms the severity linked with nonsense variants. The current report adds to the literature with a novel homozygous *ABCG8* variant (p.Pro231Thr, exon 5) found in both siblings. Such variants may contribute to a less severe hematologic phenotype compared to truncating mutations, although individual variability is considerable.

### Hematological manifestations

The clinical features of sitosterolemia encompasses a spectrum ranging from being totally asymptomatic to involvement of multiple organ systems. Hematologic abnormalities in sitosterolemia include hemolytic anemia and bleeding episodes as a result of dysfunctional macrothrombocytes. Stomatocytosis is a morphological consequence of abnormal membrane lipid composition, not the direct cause of hemolysis. Hemolysis more likely arises from the incorporation of plant sterols into red cell membranes, rendering them rigid (less deformable) and more susceptible to splenic clearance. Splenic lipid accumulation and sequestration may also contribute to both anemia and thrombocytopenia [11]. Hemolytic anemia with stomatocytes and macrothrombocytopenia was a consistent finding in the present report. Hb ranged from 5.7 g/dL to 14.9 g/dL and platelet counts ranged from 50,000/µL to 66,000/µL, as reported by Zhao et al. [10] and Risinger et al. [9], respectively. Blood smears revealed stomatocytes in both the siblings in the current report. Macrothrombocytopenia, along with elevated mean platelet volume and platelet distribution width, further supports the morphological abnormality in typical cases [2]. These findings were contrasted with dehydrated stomatocytosis; our patients presented with normal iron profiles and no evidence of hepatic steatosis, thereby ruling out potential confounders for hemolysis or hyperferritinemia.

### Bleeding tendency

Similarly, attributing hemorrhage solely to dysfunctional macrothrombocytes underestimates the complexity of the platelet phenotype. Mechanistic studies by Kanaji et al. show that phytosterol accumulation induces αIIbβ3 internalization, activation of signaling pathways, microparticle release, and calpain-mediated degradation of GPIbα and filamin A [12]. These events lead to giant platelets, defective aggregation, megakaryocytic abnormalities, and a paradoxical bleeding phenotype resembling inherited platelet function disorders.

### Therapeutic interventions and outcomes

Management of sitosterolemia primarily focuses on lowering circulating levels of plant sterols such as campesterol, stigmasterol, and β-sitosterol. This is achieved through dietary restriction of plant sterol intake, complemented by pharmacologic therapy. Oral administration of ezetimibe at a daily dose of 10 mg has demonstrated efficacy in reducing sterol absorption and improving clinical outcomes [8, 9, 10]. While in the older reports by Wang et al. [7] cholestyramine was used, newer cases, including the present report, demonstrated effective hematologic recovery with ezetimibe (10 mg/day). A comparison of hematological parameters and outcomes of the present cases with previously published reports exhibiting pronounced hematological features is summarized in Table 2.

**Table 2:** Comparison of hematological parameters and outcomes of the present cases with previously published reports exhibiting pronounced hematological features.

Source / Year	Number of Patients (Age range / Male: Female ratio)	Genetic Mutation (Location)	Hb (g/dL)	Platelet Count (/µL)	Treatment
Present Report	2 siblings, 12- and 10-year-old females	*ABCG8* - homozygous (p.Pro231Thr exon 5 in both)	7.6 / 9.9	80 / 105	Sterol-restricted diet + Ezetimibe 10 mg, Sterol-restricted diet
**Wang et al.** [7]	13 (23- 61 years, 1: 1.16)	*ABCG5/ABCG8* - homozygous nonsense mutations (e.g., C18802T in exon 1, C20896T in exon 10, G19839A in exon 9, and ISV713G>A splice site), compound heterozygous variants in *ABCG5* (A20883G intron 9 and C20896T exon 10), and in *ABCG8* (del43683–43,727,del43866C-43867G/ins43866T in exon 3, G18729A exon 11, and 10 bp insertion exon 11)	Mean 10.7	Mean 33,520	Sterol-restricted Diet, + cholestyramine
**Desai et al.** [8]	4 patients (7–29 years, 1:1)	*ABCG5* - 3 homozygous (p.R243X, p.R408X, p.G111E and 2 heterozygous: p.R408X and p.R419C)	Mean 7.93	Mean 19,250	Sterol-restricted diet ± Ezetimibe 10mg
**Risinger et al.** [9]	1 19-year-old male	Presumed - *ABCG8* compound heterozygous (c.63_63+53del and c.547del over exon1 and intron1 boundary)	14.9	66	Sterol-restricted diet + Ezetimibe 10mg
**Zhao et al.** [10]	1 8-year-old male	*ABCG5* - homozygous non sense (p.Arg446Ter in exon 10)	5.7	50	Sterol-restricted diet + Ezetimibe 10mg

This report describes only two patients from the same family, which limits how broadly the findings can be applied. Due to limited resources, plant sterol levels could not be measured, and the long-term effects of treatment are still unclear. A novel missense mutation (p.Pro231Thr) was identified in the present report, however its functional impact biochemically is not validated. Even so, its presence in both siblings and inheritance pattern support its likely role in the disease. Even with the small sample size, this case contributes valuable insight to the limited pediatric literature on sitosterolemia in India. These findings highlight the need to consider sitosterolemia in cases of Coombs-negative hemolytic anemia and unexplained macrothrombocytopenia. Family screening initiated by the index case is crucial; in this report, this investigation facilitated the early diagnosis and management of an asymptomatic sibling, underscoring the clinical utility of timely genetic testing. Despite being a potentially treatable condition, it remains largely underdiagnosed due to its nonspecific clinical features and the lack of routine plant sterol testing. Early molecular confirmation is of utmost importance to avoid diagnostic delays and to initiate targeted interventions, such as dietary sterol restriction and pharmacologic inhibition of sterol absorption using ezetimibe, which can result in substantial clinical improvement and prevention of long-term complications.

## Declaration of patient consent

The authors certify that they have obtained all appropriate patient consent.

## Conflicts of interest

There are no conflicts of interest
